# Biomechanical Impacts of Toe Joint With Transfemoral Amputee Using a Powered Knee-Ankle Prosthesis

**DOI:** 10.3389/fnbot.2022.809380

**Published:** 2022-03-16

**Authors:** Shawanee' Patrick, Namita Anil Kumar, Woolim Hong, Pilwon Hur

**Affiliations:** ^1^Human Rehabilitation Group, Texas A&M University, Mechanical Engineering, College Station, TX, United States; ^2^Gwangju Institute of Science and Technology, Department of Mechanical Engineering, Gwangju, South Korea

**Keywords:** prosthesis, flexible foot, kinetics, kinematics, powered prosthesis, symmetry, transfemoral, biomechanics

## Abstract

Transfemoral amputees are currently forced to utilize energetically passive prostheses that provide little to no propulsive work. Among the several joints and muscles required for healthy walking, the ones most vital for push-off assistance include the knee, ankle, and metatarsophalangeal (MTP) joints. There are only a handful of powered knee-ankle prostheses (also called powered transfemoral prostheses) in literature and few of them comprise a toe-joint. However, no one has researched the impact of toe-joint stiffness on walking with a power transfemoral prosthesis. This study is aimed at filling this gap in knowledge. We conducted a study with an amputee and a powered transfemoral prosthesis consisting of a spring loaded toe-joint. The prosthesis's toe-joint stiffness was varied between three values: 0.83 Nm/deg, 1.25 Nm/deg, and infinite (rigid). This study found that 0.83 Nm/deg stiffness reduced push-off assistance and resulted in compensatory movements that could lead to issues over time. While the joint angles and moments did not considerably vary across 1.25 Nm/deg and rigid stiffness, the latter led to greater power generation on the prosthesis side. However, the 1.25 Nm/deg joint stiffness resulted in the least power production from the intact side. We, thus, concluded that the use of a stiff toe-joint with a powered transfemoral prosthesis can reduce the cost of transport of the intact limb.

## 1. Introduction

There are over 1.3 million lower limb amputees in the United States alone (Ziegler-Graham et al., 2008). Over the next 50 years, this number is predicted to increase to 3.6 million (Ziegler-Graham et al., 2008). Out of this number, more than half are transfemoral (25.8 %) or transtibial (27.6 %) amputations (Dillingham et al., 2002). Transtibial (i.e., below knee) amputees do not have ankle and metatarsophalangeal (MTP) joints. Transfemoral (i.e., above knee) amputees lack a knee joint in addition to the prior listed joints. The performance with prosthesis relies on the nature of feet, the extent of actuation, comfortable fit, etc. Studies have shown that current prostheses do not account for all customer needs. Long-term use of current prosthetic feet can cause many issues such as osteoarthritis, osteopenia, and scoliosis (Gailey et al., 2008). This is due to walking asymmetries, and the missing joints and muscles required to propel the body forward during walking (Kaufman et al., 2012; Jayaraman et al., 2018). In particular, the ankle and MTP joints are vital to helping in gait progression (Stokes et al., 1979; Weerakkody et al., 2017; Honert et al., 2018, 2020). In walking the primary role of the MTP joints are to aid in stability (Fujita, 1985; Zhang et al., 2014). MTP joints were also found to be necessary to help aid in energy storage and propulsion for able bodied individuals (Goldmann and Brüggemann, 2012; Jeong et al., 2014). Although there are many prosthetic feet currently on the market, none can replicate the complex dynamics of MTP joints.

### 1.1. Evaluation of Prosthetic Feet

The most common type of prosthetic feet on the market are conventional feet (CF), and Energy Storage and Return (ESR) feet (Cherelle et al., 2014). ESR feet are claimed to be more beneficial for amputees due to a flexible keel that possibly aids with push-off during walking (Versluys et al., 2008). However, the improvements seen in energy storing and cost of transport were found to be very small (Gardiner et al., 2016). Furthermore, the push-off assistance offered by CF and ESR feet is far lesser than that of able-bodied feet. This has led researchers to attempt increasing push-off assistance by attempting to replace the action of the MTP joints by adding a toe-joint. A study by McDonald et al. (2021) added a toe-joint to a passive ankle-foot prosthesis and found no significant differences in kinetics and kinematics. However, a passive foot with a flexible toe-joint by Honert et al. (2020) showed there was a difference using a custom foot with a wider base, longer arch, and a toe-joint. So, there is no consistency in the benefits of passive feet with flexible toes. While these studies only looked at the impact of a toe-joint on transtibial amputees, the impact on transfemoral amputees is yet to be explored.

### 1.2. Powered Prosthetic Ankles

Lower limb prostheses are either powered or passive, with the latter being more popular. There is currently only one powered prosthetic ankle on the market, the BiOM. This powered ankle has significantly improved ankle power and cost of transport for transtibial amputees (Ferris et al., 2012; Herr and Grabowski, 2012). Several other powered prostheses have been explored in the research community (Sup et al., 2008; Grabowski et al., 2010; Zhu et al., 2014; Lenzi et al., 2017; Quintero et al., 2018). There has been some work on combining powered ankles with toe-joints (Zhu et al., 2014). This study's foot design has an active toe-joint and active ankle, which produced more symmetric walking than passive feet in terms of joint angles and GRF. However, none have investigated the impact of toe-joints on the performance of powered knee-ankle prosthesis. Due to the positive impact of the MTP joint and powered ankles for transtibial amputees, we must study whether transfemoral amputees also stand to benefit from such joints. Given that transfemoral amputees makeup almost 26% of the ever growing lower limb amputee community, it is of paramount importance that we address this gap in knowledge (Dillingham et al., 2002). When researching powered prostheses, we cannot limit our observations to the impact of the toe-joint alone. We must also consider the nature of the prosthesis control, which affects how the user interacts with the device as well as kinetic and kinematic outcomes.

This study analyzed the use of an actuated knee-ankle prosthesis with a toe-joint for transfemoral amputees. We explore how three different toe-joint stiffnesses impact spatiotemporal measures, kinetics, and kinematics. Our hypothesis is that the lower stiffness spring will provide less push-off power during walking compared to a stiffer and rigid stiffness foot. The article is organized as follows. Section 2 presents the equipment overview, experiment setup, protocol, and data processing methods. The results are presented in Section 3 followed by the discussion in Section 4. The final section consists of our concluding remarks.

## 2. Methods

### 2.1. Equipment Overview

**Figure 1:**
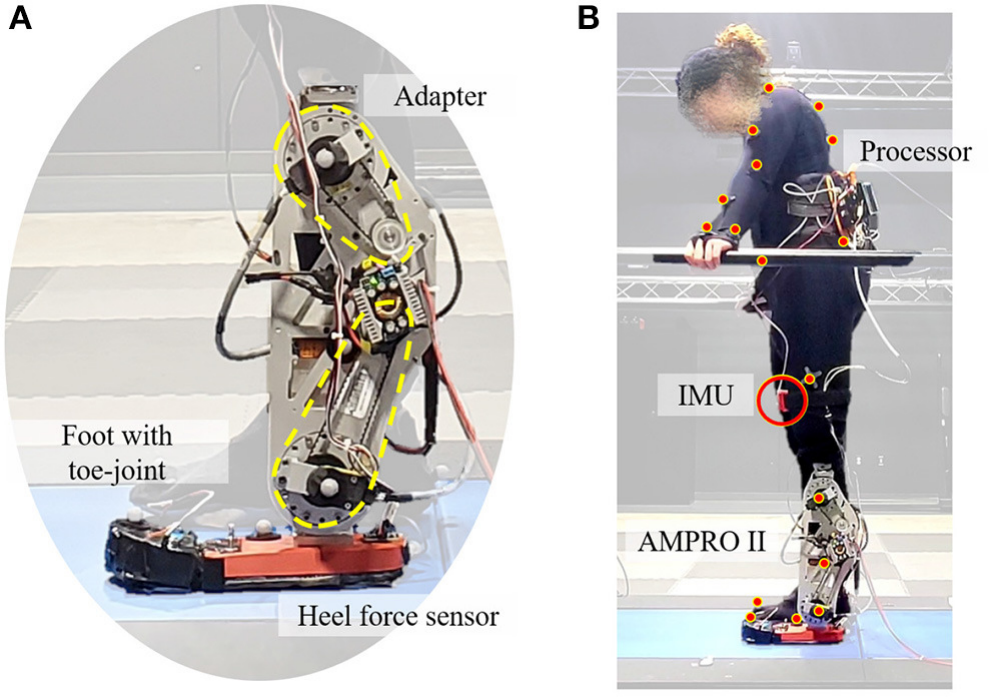
Experimental setup: **(A)** is the powered transfemoral prosthesis, AMPRO II, **(B)** shows the amputee walking with AMPRO II in a motion capture environment.

**Figure 2:**
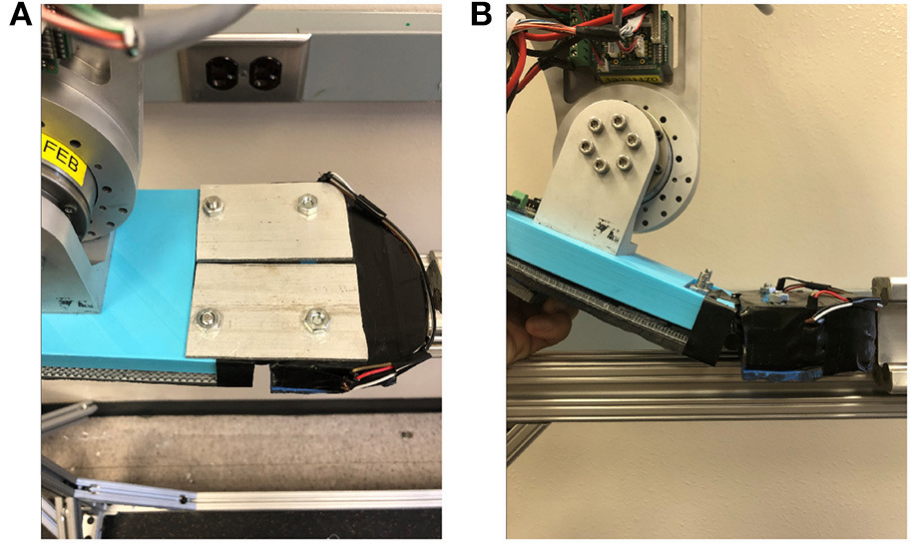
**(A)** AMPRO II with locked rigid Foot, **(B)** AMPRO II with Flexed foot.

This study utilized AMPRO II, a powered knee and ankle prosthesis (Figure 1), which is operated by a microprocessor (element14, BeagleBone Black) that controls actuated ankle and knee joints. The prosthesis is equipped with a 3D printed foot with an MTP joint (Figure 2). The toe-joint was equipped with an leaf spring utilizing spring steel sheets. The stiffness of the joint was varied by varying the number of spring steel sheets. The lowest stiffness (0.83 Nm/deg) was found to be within 0.01 Nm/deg of the average estimated stiffness of the MTP joint during able bodied walking (Mager et al., 2018). Furthermore, a force sensor (Tekscan, FlexiForce A502) placed under the heel helps detect heel-strike, while an Inertial Measurement Unit (SparkFun Electronics, MPU 9150) affixed to the user's thigh measures the thigh angle. This thigh angle is used to estimate the user's walking progress and thereby the user's intent (Hong et al., 2021). This powered prosthesis is controlled using impedance control during the stance phase and trajectory tracking control during the swing phase. The stance phase is divided into 3 states: (i) heel-strike to flat-foot, (ii) flat-foot to heel-off, and (iii) heel-off to toe-off. The torque generated by the impedance control strategy is given by


(1) $$\tau =K(\theta -\theta_{ref})+D\dot{\theta }$$

where *K* and *D* are the joint stiffness and damping parameters. The term θ_*ref*_ is the joint's reference or equilibrium angle. θ and $\overset{∙}{\theta }$ are the joint's instantaneous position and velocity, making the impedance control scheme very responsive to the user's kinematics. The user can increase the amount of generated torque by deviating more from θ_*ref*_. Thus, the user has some control over the generated torque or push-off assistance (Lawson et al., 2014). Both *K* and *D* varied as polynomials of the user's walking progression, while θ_*ref*_ was constant during each state. These parameters were found through a data-driven approach wherein a least squares optimization minimized the difference between Equation (1) and healthy human walking joint torque. The optimized parameters vary such that each joint can dampen, support, and propel the user in accordance with the walking progress. For example, the ankle's stiffness increases as the user progress from heel-strike to heel-off, with the peak occurring at max push-off torque. More details on the optimization and the control strategy can be found in Anil Kumar et al. (2022).

All experiments were conducted in a motion capture lab that utilizes 44 motion capture cameras (Vantage, Vicon Motion Systems Ltd., Oxford, UK) and a force-sensing tandem instrumented treadmill (AMTI, Watertown, MA, USA). The motion capture camera was collected at 100 Hz and the treadmill force plate data were collected at 1,000 Hz.

### 2.2. Experiment Overview

This study had one participant who is a unilateral transfemoral amputee (female, 164 cm, 66 kg w/o prosthesis). She currently utilizes an X3 microprocessor Knee (Ottobock, Duderstadt, Germany) with a Freedom Runaway Foot (Ottobock, Duderstadt, Germany). In order to collect motion capture data, the full-body plug-in gait marker set from Vicon Nexus was used (Vicon Motion Systems Ltd., Oxford, UK).

#### 2.2.1. Protocol

The participant underwent eight practice sessions to get accustomed to the powered prosthesis and different feet. The participant was most comfortable walking at a speed of 0.67 m/s. The participant walked with three joint stiffness conditions: 0.83 Nm/deg, 1.25 Nm/deg, and Infinite (Rigid). Motion capture and force plate data were collected for each foot variation. Each walking trial lasted 90 s with 10 min breaks between foot changes. The participant was allowed to take a longer rest if requested.

### 2.3. Data Processing

All post-processing was done in Vicon Nexus and Visual3D (C-Motion, Germantown, MD, USA). The marker trajectories and the force data were filtered in Vicon Nexus with a low-pass third-order butter worth filter at 10 and 20 HZ, respectively. The hip, knee, and ankle joint angle, moment, and power were calculated in the sagittal plane using the Visual3D software.

The following spatiotemporal metrics were collected using marker data and force data: total step length, step time, swing time, and stance time. These were collected for both the intact and prosthetic limbs. Step length was calculated to be the total distance from heel-strike of one foot to heel-strike of the opposite foot. Step time is the time from heel-strike of one foot to heel-strike of the opposite foot. Swing time is measured to be the time from toe-off to heel-strike. Stance time is measured to be the time from heel-strike to toe-off.

To see how much the stiffness impacts symmetry between the intact and prosthesis side, the symmetry index (SI) was calculated for each of the measured spatiotemporal metrics. Ideally, the step time, swing time, and step length should be relatively close between both limbs. The higher the deviations are, the less symmetric the walking (Robinson et al., 1987). We will use Equation (2) where *X*_*P*_ is the spatiotemporal metric on the prosthesis side and *X*_*I*_ is the metric on the intact leg. If this value is negative, the dominant leg for the corresponding metric is the intact leg. The desire is for this value to be as close to zero as possible. The values fall between −100 and 100.


(2) $$\begin{array}{r} SI=\frac{\left(X_{P}-X_{I}\right)}{0.5\left(X_{P}+X_{I}\right)}*100 \end{array}$$

For all spatiotemporal metrics, one-way repeated-measures ANOVA was done using python's statsmodel library with α = 0.05. If this showed significant impact of toe-joint stiffness, two-tailed paired *t*-tests were conducted for all combinations of toe-joint stiffness using python's scipy library with α = 0.05.

## 3. Results

### 3.1. Spatiotemporal Data

On the prosthesis side, there was a significant impact of toe-joint stiffness on step time (*p* < 0.001), stance time (*p* = 0.001), swing time (*p* = 0.001), and step length (*p* = 0.02). Mean step time with the 0.83 Nm/deg joint stiffness, was shown to be significantly greater than with the 1.25 Nm/deg and rigid joint stiffness (*p* < = 0.003 for both comparisons). This is also true for step length (*p* < 0.03), stance time (*p* < = 0.001), and swing time (*p* < 0.02) metrics.

**Figure 3:**
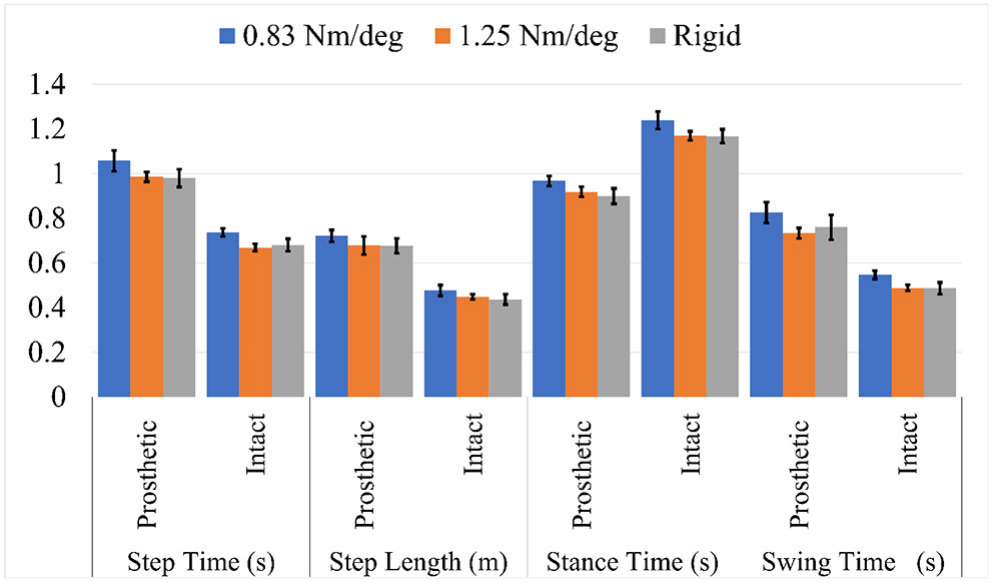
Spatiotemporal metrics for intact and prosthesis legs.

On the intact side, there was a significant impact of toe-joint stiffness on step time (*p* < 0.001), stance time (*p* < 0.001), swing time (*p* < 0.001), and step length (*p* < 0.001). Per pairwise *t*-tests, step time (*p* < 0.001), swing time (*p* < 0.001), and stance time (*p* < 0.003) were significantly greater with 0.83 Nm/deg joint stiffness than those with the 1.25 Nm/deg and rigid joint stiffness. The aforementioned *p* values are for both pairwise comparisons: 0.83 vs. 1.25 Nm/deg and 0.83 Nm/deg vs. rigid. This can be seen in Figure 3.

**Figure 4:**
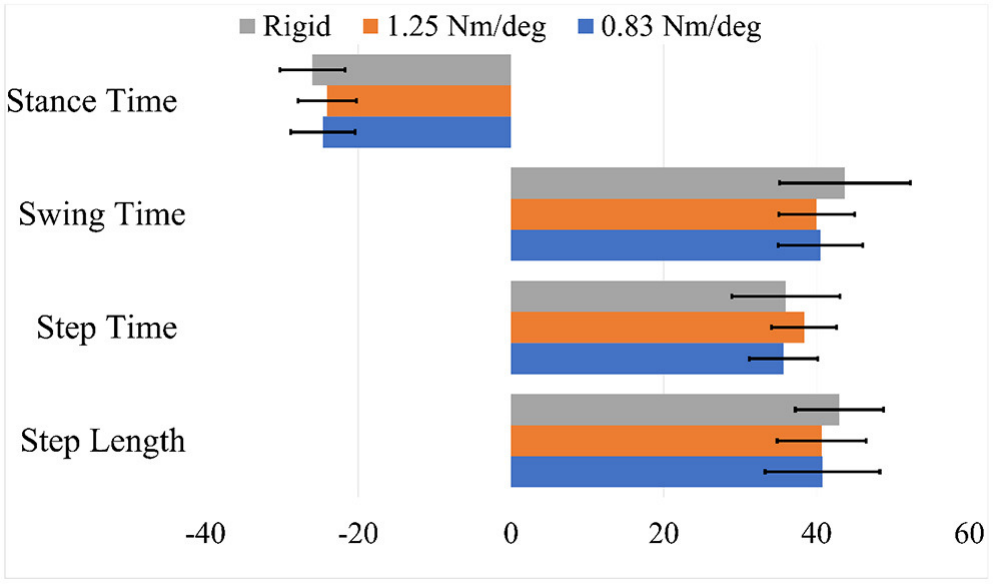
Symmetry index (SI) for spatiotemporal metrics.

Although the step lengths and step times were significantly greater while using the 0.83 Nm/deg joint stiffness, the SI index for all spatiotemporal values was found not to vary significantly with toe-joint stiffness (*p*>0.34). The 1.25 Nm/deg joint stiffness was found to be slightly more symmetric for stance and swing time, but these differences were not found to be significant (Figure 4).

### 3.2. Kinetics and Kinematics

**Figure 5:**
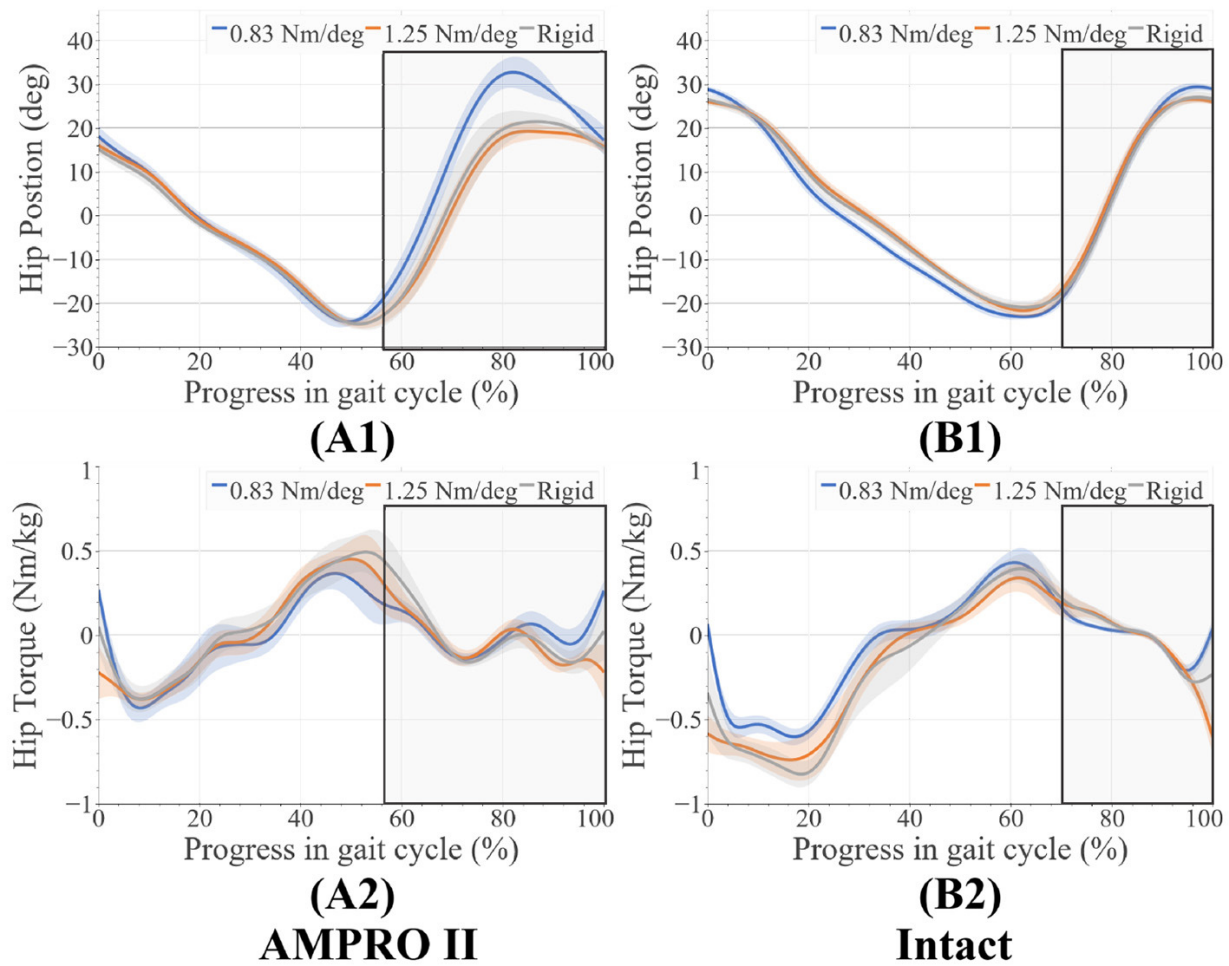
**(A1)** Hip angles on prosthesis side, **(A2)** hip moments on prosthesis side, **(B1)** hip angles on intact side, **(B2)** hip moments on intact side, average swing phase for each case is boxed in the gray.

With the 0.83 Nm/deg joint stiffness, the hip flexion at the end of the swing was 10 degrees greater than the rigid joint stiffness and 12 degrees greater than the 1.25 Nm/deg joint stiffness (Figure 5A1). The maximum hip torque increased with stiffness (Figure 5A2). Hip angles and hip moments on the intact side (Figure 5B1,5B2) had similar trends between stiffnesses.

**Figure 6:**
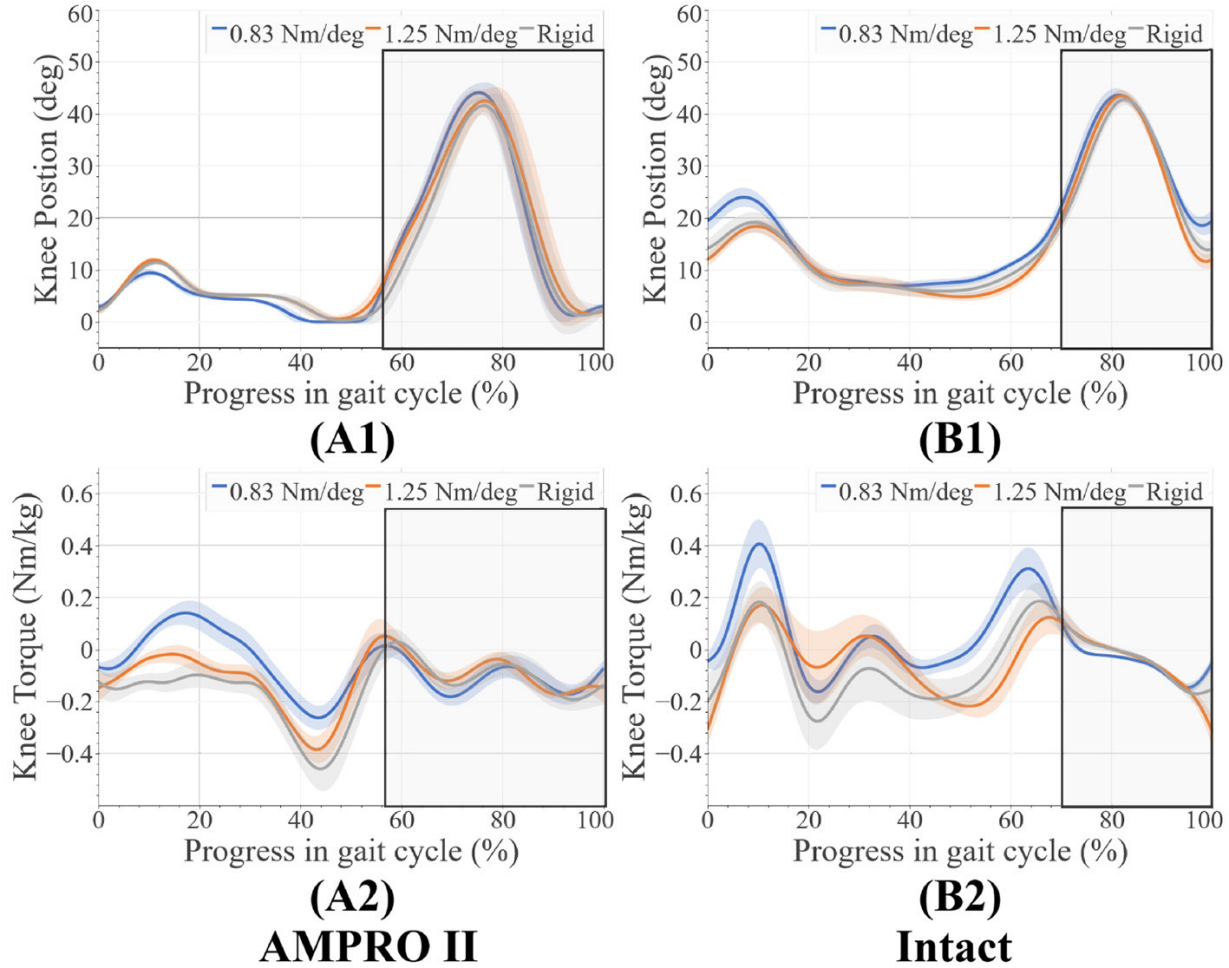
**(A1)** Knee angles on prosthesis side, **(A2)** knee moments on prosthesis side, **(B1)** knee angles on intact side, **(B2)** knee moments on intact side, average swing phase for each case is boxed in the gray.

**Figure 7:**
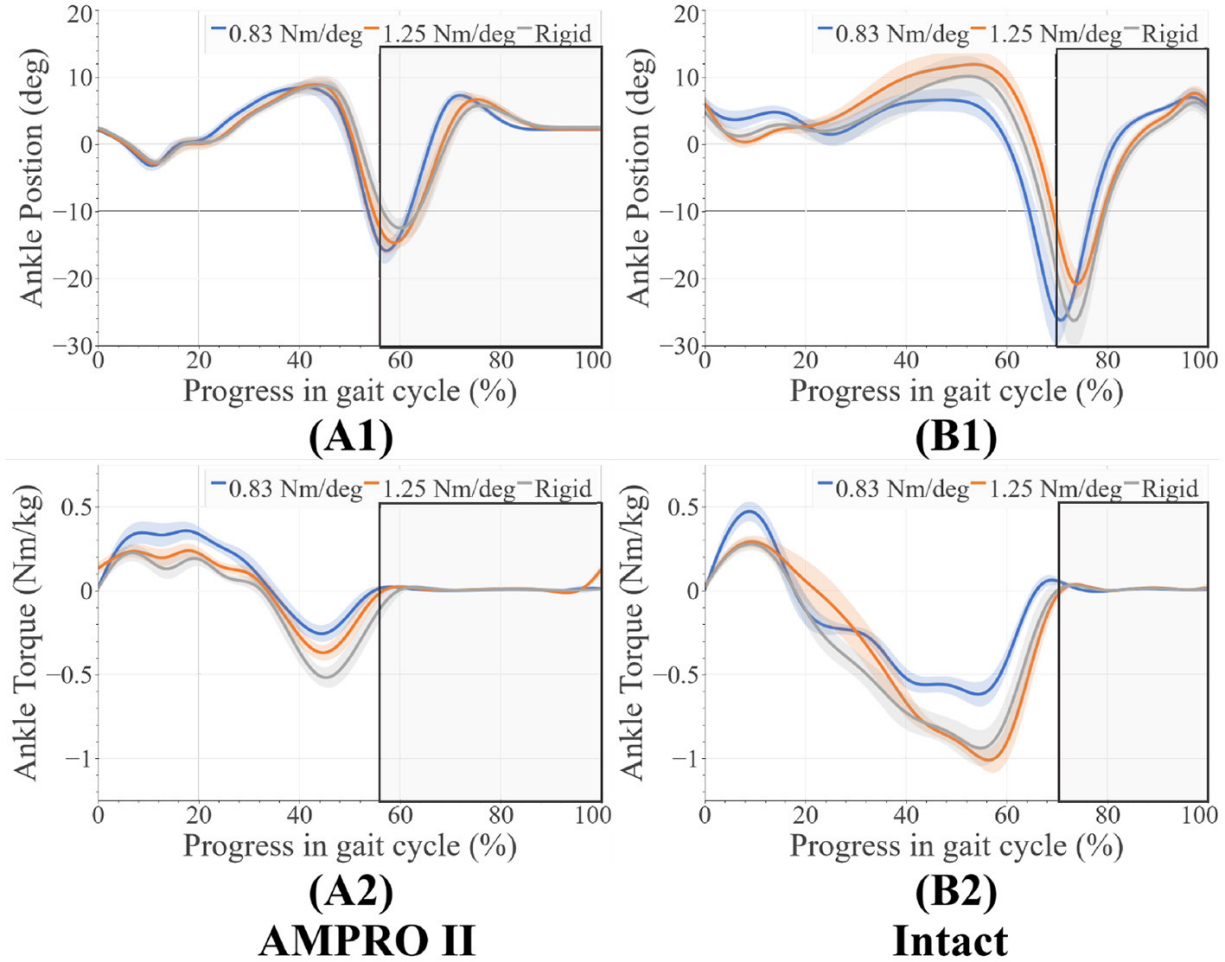
**(A1)** Ankle angles on prosthesis side, **(A2)** ankle moments on prosthesis side, **(B1)** ankle angles on intact side, **(B2)** ankle moments on intact side, average swing phase for each case is boxed in the gray.

There were very few changes in knee range of motion for different toe stiffness. On the prosthesis side, there was greater flexion torque in early stance when using the 0.83 Nm/deg joint stiffness compared to the 1.25 Nm/deg joint stiffness (+0.13 Nm/kg) and rigid stiffness (+0.20 Nm/kg) (Figure 6A2). When using the 0.83 Nm/deg joint stiffness less extension torque early before push off compared to the 1.25 Nm/deg joint stiffness (−0.15 Nm/kg) and rigid stiffness (+0.22 Nm/kg) (Figure 6A2). There were also higher peak knee flexion moments for the 0.83 Nm/deg joint stiffness (Figure 6B2) on the intact side. Range of motion of the knee for both the intact side (±2 degrees) (Figure 6B1) and prosthesis side (±3 degrees) (Figure 6B2) differed very little between foot stiffnesses. On the prosthesis side, the ankle range of motion was very similar (± 2 degrees) (Figure 7A1). The ankle moment on the prosthesis side decreased with stiffness at the beginning of stance and decreased with stiffness before push off (Figure 7A2). The intact ankle resulted in more dorsiflexion at the end of stance for the 1.25 Nm/deg (+ 5 degrees for 0.83 Nm/deg joint stiffness, +1.75 degrees for Rigid joint stiffness) (Figure 7B1). However, both the rigid and the 0.83 Nm/deg joint stiffness had approximately 5.4 degrees more plantar flexion than the 1.25 Nm/deg foot (Figure 7B1). The plantar flexion ankle moment before push-off with the 0.83 Nm/deg joint stiffness was less than the 1.25 Nm/deg and rigid joint stiffness by 0.5 and 0.4 Nm/Kg, respectively (Figure 7B2).

**Figure 8:**
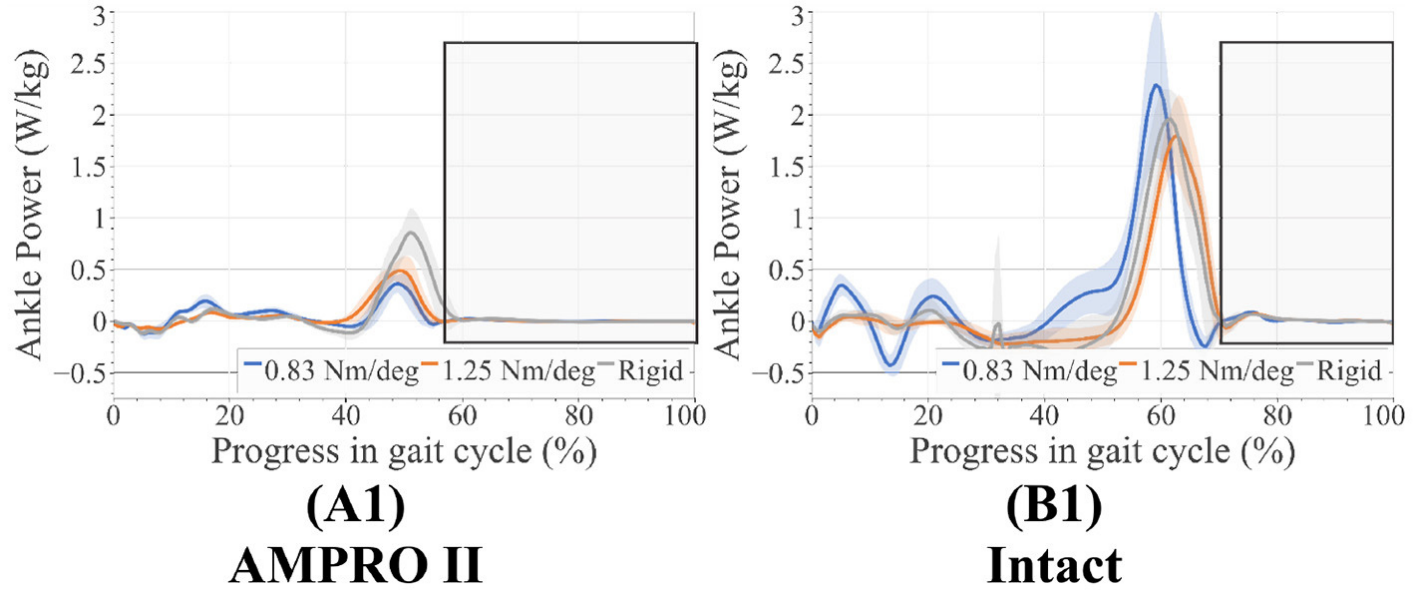
**(A1)** Ankle power on prosthesis side, **(B1)** ankle power on intact side, average swing phase for each case is boxed in the gray.

As seen in (Figures 8A1, 8B1, and 9), peak power did increase with stiffness on the prosthesis side. On the prosthesis side, 0.83 Nm/deg joint was found to produce significantly lower peak power than the 1.25 Nm/deg joint and the rigid joint (*p* = 0.0001). The rigid toe joint was found to have a significantly higher peak power than the 0.83 and 1.25 Nm/deg joint (*p* < 0.0009). On the intact side, the power decreased in the order 0.83 Nm/deg, rigid, and 1.25 Nm/deg. The rigid joint resulted in significantly higher peak power(*p* = 0.023).

**Figure 9:**
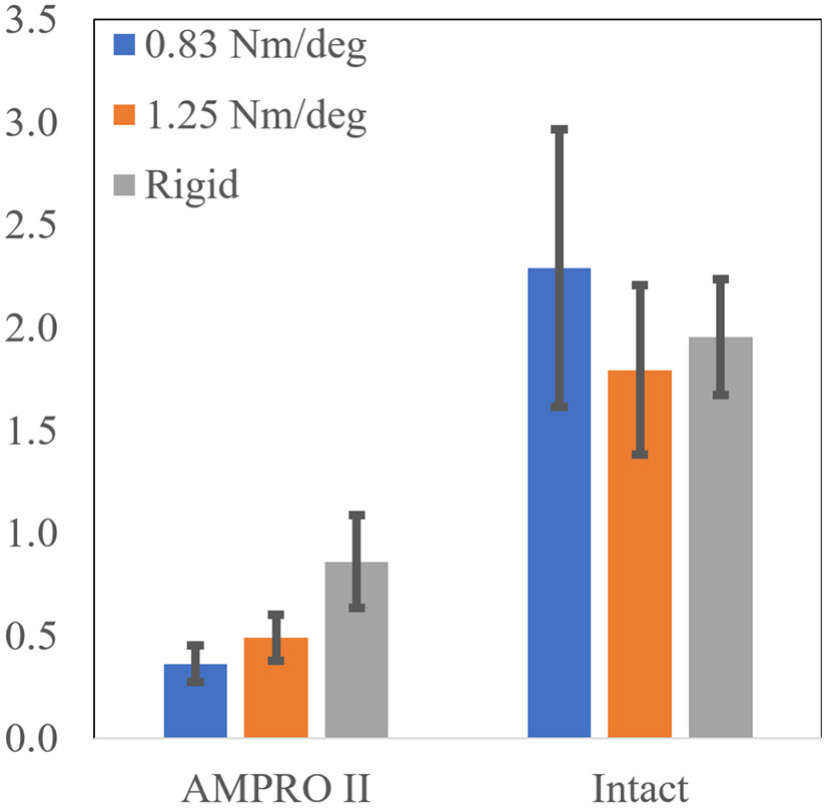
Pushoff ankle power.

## 4. Discussion

While steps with the 0.83 Nm/deg joint stiffness were longer, they did not produce a more symmetric gait. Longer stance time on the prosthesis is only beneficial if it is more symmetric. Amputees on average spend less time on the side of their prosthesis resulting in overloading of the intact leg (Nolan and Lees, 2000; Nolan et al., 2003; Cutti et al., 2018; Brandt et al., 2019). Increased time on the prosthesis side compared to other feet can seemingly be a positive thing, however, this increased time must be measured against time on the intact leg to notice if it is beneficial. Due to there being no significant differences in SI for all spatiotemporal metrics this longer stance does not provide a benefit to the user.

In the case of 0.83 Nm/deg, there were some compensatory motions that resulted. On the prosthesis side, an increased hip flexion at the end of the stance was observed. On the intact side, an increased peak knee moment, increased knee flexion and ankle dorsiflexion during heel-strike, and an increased plantarflexion before toe-off were observed. As stated in Section 2.1, deviating from the reference angle increases the generated joint torque. With the lower toe-joint stiffness, it is possible the participant is attempting to get more push-off support by elongating the step. Despite these efforts, the resulting ankle push-off torque and power were lower compared to those of 1.25 Nm/deg and rigid joint stiffnesses (Figure 9). This shows the toe-joint stiffness of 0.83 Nm/deg counters the positive impact of the powered knee-ankle prosthesis in terms of push-off assistance. In order to achieve these longer steps, the participant had to increase hip flexion during swing. The peak hip moments on the prosthesis side increased with foot stiffness. This value for the rigid stiffness was similar to the intact leg's hip moment values. This indicates more similar loading trends between the intact leg and the prosthesis as stiffness increases.

The increased knee flexion moments on the intact limb in the 0.83 Nm/deg case (Figure 6B2) indicate that there could be less stability during walking. Increased knee flexion has been correlated to knee instability during walking (Morgenroth et al., 2012). These higher moments over time have been associated with osteoarthritis (Chen et al., 2016). Using this stiffness with a powered prosthesis could counter the benefits reported in previous studies (Sup et al., 2008; Zhu et al., 2014; Lenzi et al., 2017; Quintero et al., 2018). Higher loading of the intact leg can be seen in the higher intact ankle peak power values (Figures 8, 9). The use of this foot also led to the increase of dorsiflexion moment at the beginning of stance on the intact leg, indicating an increased need for more stability at push-off. The participant was seen compensating more with their intact leg in order to walk forward with this toe-joint stiffness.

The difference in moments and power production between the prosthesis and intact leg, as well as the compensatory motion mentioned above, are some of the reasons for high incidences of arthritis in amputees (Morgenroth et al., 2011). One of the main reasons for device abandonment is discomfort (Klute et al., 2001). If users have to make these compensatory motions with a heavier powered device, they may not wish to use it. It is possible with the 0.83 Nm/deg toe-joint the participant could feel less stable during heel-strike and push-off resulting in the compensatory movements mentioned above.

These compensatory responses were not observed in the cases pertaining to 1.25 Nm/deg and the rigid foot. The latter performed best in terms of power production on the prosthesis side. This could mean that the stability provided by a locked toe-joint through stance could prove to be beneficial with some transfemoral amputees and powered devices. The rigid and 1.25 Nm/deg toe-joint scored relatively close in terms of other metrics. Although the rigid foot produced the most power on the prosthesis side, that did not result in the least power production on the intact side. The 1.25 Nm/deg case resulted in the least power production on the intact side. This shows that increased power production on the prosthesis side does not always result in lesser demand for power from the intact side. In other words, this increased power does not always minimize overloading. Given that the results with a 1.25 Nm/deg case were slightly more symmetric, this could indicate that using a toe-joint can help reduce intact limb overloading.

We postulate that the addition of a toe-joint can make a difference while walking with a powered knee-ankle prosthesis. However, a wider range of toe joint stiffness needs to be tested in order to verify if this is true. Two of the shortcomings of this study is that it involved only three stiffnesses and a single participant. Using a foot that has a stiffness greater than 1.25 Nm/deg but not fully rigid could improve the results observed in this study. Human toe joint stiffness is shown as a nonlinear trend during walking. Studies such as Um et al. (2021) have proposed using toe-joints with nonlinear stiffness. Future efforts will be directed at studying the performance of transfemoral prostheses with nonlinear stiffness toe-joints.

## 5. Conclusion

From this study, we determined the impact of using a toe-joint with a powered prosthesis for a transfemoral amputee. We tested three different stiffness. It was determined that foot stiffness is related to power production on the prosthesis leg, with higher stiffness resulting in higher push-off assistance. The lowest stiffness had the least push-off power, demanding more power production from the intact leg. Even though low stiffness (i.e., 0.83 Nm/deg) has the benefit of easy rollover during the mid-stance, it resulted in longer step time and step length and compensatory movements that could negatively impact users over time. We conclude that a toe joint with a stiffness that is too low can negatively impact the user. However, a toe joint with a suitably selected stiffness can reduce the loading on the intact leg. In addition, power production alone is not enough to indicate the effectiveness of lower limb prostheses. It is desired to look at spatiotemporal changes as well as kinetic and kinematic responses. More stiffness and toe-joint designs need to be explored with transfemoral amputees to determine if they are able to replicate the benefits of the human MTP joints.

## Data Availability Statement

The raw data supporting the conclusions of this article will be made available by the authors, without undue reservation.

## Ethics Statement

The studies involving human participants were reviewed and approved by Texas A&M IRB (IRB2015-0607F). The patients/participants provided their written informed consent to participate in this study.

## Author Contributions

SP: primary contributor to experiment design, data collection, and processing. NAK: contributed to data collection and analysis. WH: assisted with the implementation of the control scheme. PH: served as the principal investigator. All the authors contributed to writing and reviewing this article.

## Conflict of Interest

The authors declare that the research was conducted in the absence of any commercial or financial relationships that could be construed as a potential conflict of interest.

## Publisher's Note

All claims expressed in this article are solely those of the authors and do not necessarily represent those of their affiliated organizations, or those of the publisher, the editors and the reviewers. Any product that may be evaluated in this article, or claim that may be made by its manufacturer, is not guaranteed or endorsed by the publisher.
